# Insights Into the Mechanism of Action of Chlorhexidine on *Porphyromonas gingivalis*

**DOI:** 10.1155/ijod/1492069

**Published:** 2025-02-27

**Authors:** Karen Mejía, Adriana-Patricia Rodríguez-Hernández, Miryam Martínez-Hernández

**Affiliations:** ^1^BioInterphases Laboratory, Division of Graduate Studies and Research, Faculty of Dentistry, National Autonomous University of Mexico, Mexico City, Mexico; ^2^Laboratory of Molecular Genetics, Division of Graduate Studies and Research, School of Dentistry, National Autonomous University of Mexico, Mexico City, Mexico; ^3^BioInterphases Laboratory, Division of Graduate Studies and Research, School of Dentistry, National Autonomous University of Mexico, Mexico City, Mexico

**Keywords:** oral antiseptic, periodontal bacteria, virulence factors

## Abstract

Chlorhexidine (CHX) remains the most effective antiseptic in periodontal therapy, multiple reports have identified ultrastructural antibacterial effects of CHX on oral bacteria, however, little is known about its molecular mechanism of action on *Porphyromonas gingivalis*, an important pathobiont directly associated with the pathogenesis of periodontitis. A standardized suspension of *P. gingivalis* ATCC 33277 was expose to 0.20% CHX for 1 min, then counting colony forming units (CFUs) were recovered to determine the percentage of microbial inhibition. Protein extract integrity of the bacterial cells exposed to CHX was evaluated on a one-dimension sodium dodecyl sulfate polyacrylamide gel electrophoresis (1D SDS-PAGE) gel. The identification of the proteins expressed by *P. gingivalis* after its exposure to CHX was carried out by mass spectrometry (LC-MS). Exposure of *P. gingivalis* for 1 min to 0.20% CHX resulted in a 93% reduction in bacterial viability, in addition to an increase of 2.9-fold in protein expression, with the Lys gingipain protein showing the greatest increase. Exposure to 0.20% CHX 1 min on *P. gingivalis* resulted in 93% reduction in bacterial viability, in addition to inducing changes in the bacterial proteome, with an increased expression of gingipains, the main virulence factor of *P. gingivalis*.

## 1. Introduction

Periodontal diseases, mainly, gingivitis and periodontitis, comprise a group of chronic multifactorial inflammatory conditions associated with a dysbiotic biofilm that directly affects the tooth-supporting apparatus [1]. Scientific evidence indicates periodontal diseases have a considerable impact on several infectious as well as noninfectious systemic diseases such as diabetes, cardiovascular, or renal disorders [2], in addition to representing a significant, economic, healthcare, and social burden [3]. Unfortunately, despite these facts, periodontal diseases are rarely considered a health priority, particularly in low- and middle-income countries in which oral healthcare is limited due to the global burden inequalities [4].

The main etiological factor of periodontal diseases is bacteria organized in biofilms, which colonize the oral cavity heavily. When dental biofilms are not periodically removed, dysbiotic microbiome can result, which, along with dysregulated inflammation of the host periodontal tissues, leads to the growth of specific microorganisms, such as *Porphyromonas gingivalis*, within the biofilm that plays a causal role in the development of periodontitis [5, 6].

*Porphyromonas gingivalis* is a Gram-negative, strictly anaerobic, and nonmotile bacteria [7], which successfully colonizes the oral epithelium; it is considered a biofilm later colonizer [8, 9]. Through secreting a set of virulence factors, *P. gingivalis* can invade periodontal tissues, leading to dysregulation of the host's immune response and inflammation [10]. The major virulence factors of *P. gingivalis* include lipopolysaccharides (LPSs), capsule, fimbriae, and gingipains, through them, it can invade cells and tissue, avoiding immune surveillance [11]. Once inside the cell, *P. gingivalis* secretes the enzyme ATP-hydrolase to evade ATP-dependent apoptosis [7, 12]. *Porphyromonas gingivalis* can secrete a broad variety of proteases and generates the metabolic energy for its survival in deep periodontal pockets by fermenting amino acids released [7]. Proteases called gingipains represent the main virulence factor of *P. gingivalis*.

In general terms, the prevention of periodontal diseases involves the mechanical removal of biofilms and preventing their maturation with periodontal pathogenic colonizers [13]. However, it has been reported that due to a lack of motivation, insufficient compliance, and inadequate patient skill, the mechanical control of dental biofilm using toothbrushes and dental floss may not be enough to prevent the disease [14]. Hence, to supplement mechanical removal, mouthwashes formulated with antimicrobial agents have shown high levels of complementary effectiveness [15].

The use of chlorhexidine (CHX) as a mouth rinse for inhibiting dental biofilms was first investigated in 1969 [16], since then, it has remained the gold standard against which other newer mouth rinses are measured [17, 18]. Several decades of research have proven that 0.20% CHX prevents and controls dental biofilm formation and breaks up the existing one [19], as well as inhibits and reduces gingivitis [20–22]. CHX exerts its antimicrobial effects by damaging bacterial cytoplasmic membranes and allowing their contents to leak out. Early studies showed that CHX inhibits the glycosidic and proteolytic activities of different oral bacteria, including *P. gingivalis* [23], in addition to reducing the adherence of *P. gingivalis* to epithelial cells [24]. Aside from inhibiting several matrix metalloproteinases, CHX inhibits the collagenase activity of *P. gingivalis* [25, 26]. At ultrastructural level, 0.20% CHX damages the bilipid bacterial membranes, causing the membrane to lose its structural integrity [27]. While there are numerous reports documenting the inhibitory effects of CHX on *P. gingivalis*, little is known about the mechanism of action of CHX on *P. gingivalis* at a molecular level. Therefore, the present study aims to identify changes in the proteome of *P. gingivales* derived from their exposure to 0.20% CHX.

## 2. Material and Methods

### 2.1. Bacterial Culture and CHX Exposure

Pure cultures of *P. gingivalis* ATCC 33277 were used for bacterial inhibition assay and total protein analyses. The methods used to grow the bacterial strain under anaerobic conditions (10% CO_2_, 10% H_2_, and 80% N_2_) at 35 ± 1°C have been previously described [28]. Briefly, bacterial growth from 5 to 7-day cultures was harvested from enriched *Mycoplasma* agar plates (Becton Dickinson [BD], Sunnyvale, CA, USA) supplemented with 5% defibrinated sheep blood, 5 µg/mL hemin (Sigma Chemical Co., UK) and 0.3 µg/mL menadione (Sigma Chemical Co., UK). Culture flasks of enriched *Mycoplasma* broth were added with a standardized bacterial suspension, measure by optical density *λ* = 600 nm with spectrophotometer Eppendorf adjusted to one to obtain 1 × 10^9^ cells per mL. Then, 1:100 dilutions were performed to a final concentration of 1 × 10^6^ cells per mL. Bacterial cells were grown in triplicates until the early stationary phase (~18–24 h of growth); afterward, the bacteria culture flasks were supplemented with CHX digluconate solution (Sigma Chemical Co., UK) to a final concentration of 0.20% during 1 min, under constant agitation. Bacteria culture flasks that were not supplemented with CHX were considered as control. Planktonic cells were plating on supplemented *Mycoplasma* plates and incubated anaerobically for 4–5 days at 35 ± 1°C, to recover the cell viability by counting the numbers of colony forming units (CFUs), after the 1 min, exposure to 0.20% CHX. Another set of planktonic cells was collected for proteome analyses.

### 2.2. Total Protein Extraction

Briefly, the planktonic cells of *P. gingivalis* ATCC 33277 either exposed and nonexposed to 0.20% CHX was collected and centrifugated at 8000 × *g*, 4°C, for 17 min, and the supernatant was discarded. The resulting pellets were suspended in 6 mL of phenol pH 8.8 and 5 mL of extraction buffer (100 mM tris-HCl, pH 8.8, 10 mM EDTA, 900 mM sucrose, and 0.4% of 2-betamercaptoethanol). Bacterial cells were disrupted by applying five pulses of 1 min in a polytron alternated for 1 min on ice; after the five pulses, the samples were kept on ice for 10 min and then centrifuged at 4000 rpm for 30 min, the supernatants were recovered in a 50 mL tube and 25 mL of 100 mM ammonium acetate in methanol was added, shaken, and left to stand 16 h at −20°C. The pellets were then centrifuged at 4000 rpm for 30 min, and the supernatants were discarded. Then, the pellets were washed two times with 5 mL of ammonium acetate in methanol and centrifuged at 4000 rpm for 10 min. Later, the pellets were additionally washed two times with 80% acetone and with 70% ethanol. Finally, the pellets were resuspended in 700 µL of isoelectric focusing buffer. Subsequently, the integrity of the proteins extracted was analyzed in a one-dimension sodium dodecyl sulfate polyacrylamide gel electrophoresis (1D SDS-PAGE) gel. The protein concentration was determine calorimetrically using the Pierce BCA Protein Assay Kit (Thermo Scientific, Rockford, IL).

### 2.3. Protein Identification by Mass Spectrometry (MS)

The samples were processed as described previously by Gutierrez-Sanchez et al. [29]. Briefly, gel bands were selected on corresponding gels and cut into very small pieces. Subsequently, they were treated with 5% (*v*/*v*) acetic acid and 50% (*v*/*v*) methanol for 12 h. Faded gels were washed with deionized water and incubated for 15 min in 100 mM ammonium bicarbonate. Subsequently, ditiotreitol was added at 50 mM for 45 min as a reducing agent. After time, 30 mM iodoacetamide was added and incubated for 2 h at room temperature in the dark. Pieces were subsequently washed three times with 100 mM of ammonium bicarbonate and dehydrated with 100% acetonitrile in vacuum. Digestion was carried out by adding 30 μL of modified porcine trypsin solution at 20 ng/μL (Promega) in 50 mM ammonium bicarbonate, followed by incubation for 24 h at 37°C. The resulting peptides were extracted twice in 50% (*v*/*v*) acetonitrile and 5% (*v*/*v*) formic acid for 30 min with sonication. The volume obtained was diminished by evaporation in a vacuum centrifuge and adjusted to 20 μL with 1% (*v*/*v*) formic acid. Mass spectrometric analysis of the peptides was carried out using an integrated nano-LC-ESI MS/MS system: quadrupole/time of light, synapt G2 high-definition mass spectrometer (Waters Corporation) equipped with a NanoLock Spray ion source. The instrument was coupled online to a NanoAcquity ultra-performance liquid chromatography (UPLC; Waters Corporation). Two percent of acetonitrile in Milli Q water with 0.1% formic acid (mobile phase A) and 98% acetonitrile in Milli Q water with 0.1% formic acid (mobile phase B) were used as a binary solvent system in a C18 UPLC symmetric capture column (5 µm, 180 µm × 20 mm; Waters Corporation). The samples were desalted, concentrated, and washed with 100% of the mobile phase A at a flow rate of 15 μL/min. Then, after 3 min, the capture column was changed in line with an analytical column. The peptides were separated on a C18 UPLC column (1.7 µm, 75 µm × 100 mm; Waters Corporation) with the use of linear gradient of 40% B over a period of 30 min at a flow rate of 0.3 μL/min, followed by a 98% wash of mobile phase B. Data processing was performed using the global ProteinLynx version 2.5.1 server and software (Waters Corporation) with a Protein Lynx Global Server (PLGS) (Waters Corporation). PLGS score of >95% confidence interval was accepted as correct. The UNIPROT database (https://www.uniprot.org) was searched. Peptides were matched with the theoretical peptides of reported proteins from *P. gingivalis* ATCC 33277.

## 3. Results

### 3.1. Inhibition of *Porphyromonas gingivalis* After Exposure to 0.20% CHX

**Figure 1:**
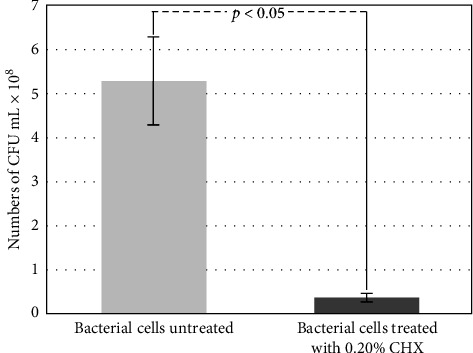
Effect of 0.20% chlorhexidine (CHX) on *Porphyromonas gingivalis* colony forming units (CFUs) counts after 60 s of exposure.

The inhibitory capacity of 0.20% CHX on *P. gingivalis* was evaluated by counting CFUs per mL (Figure 1).

For bacterial cells that were not exposed to CHX (control), 5.2 ± 0.10 × 10^8^ CFUs were quantified, while in the experimental group which was exposed for 1 min to 0.20% CHX, 3.8 ± 0.08 × 10^7^ CFUs were quantified, this difference was statistically significant (*p* < 0.05). These results indicate that exposure of bacterial cells to CHX at a concentration of 0.20% induced bacterial inhibition of 92.7%.

### 3.2. Identification of the Changes in the Proteome of *Porphyromonas gingivalis* Derived From its Exposure for 1 min to 0.20% CHX

#### 3.2.1. Electrophoretic Analysis

**Figure 2:**
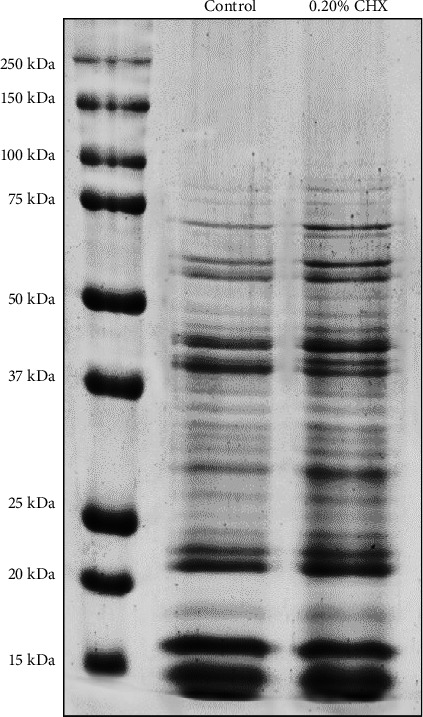
The electrophoretic pattern was obtained from the one-dimension sodium dodecyl sulfate polyacrylamide gel electrophoresis (1D SDS-PAGE) gel separation of *Porphyromonas gingivalis* protein extracts without exposure (lane 1) and 1-min exposure to 0.20% chlorhexidine (CHX; lane 2).

A 1D SDS-PAGE evaluation was made to confirm the integrity of protein extracts derived from *P. gingivalis* cells treated for 1 min with 0.20% CHX (experimental) and bacterial cells that were not exposed to CHX (control) (Figure 2). The average amount of proteins corresponding to the control group was 3.34 μg/μL, while in the experimental group was 4.26 μg/μL.

In the protein extract corresponding to bacterial cells exposed to 0.20% CHX (lane 2), it is possible to observe a higher protein expression compared to the control sample (lane 1), the bands in lane 2 are visibly thicker with respect to those of the control lane. Besides, it is possible to observe that for control and experimental groups, most protein bands are grouped in the molecular weight interval of 15–75 kDa, where more than 26 bands can be identified.

### 3.3. Identification of the *Porphyromonas gingivalis* Proteome Changes After CHX 0.20% Exposure

**Figure 3:**
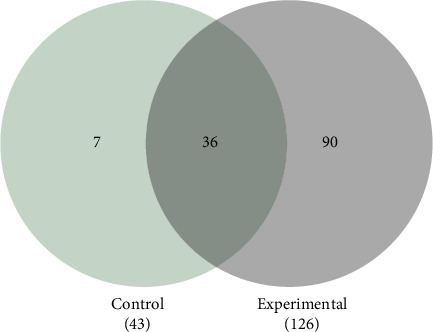
Differential expression of proteins in the proteome of the *Porphyromonas gingivalis* cells under different conditions, treated and untreated to 0.20% CHX. Venn diagram giving an overview of the numbers of consistently or uniquely identified proteins under the experimental conditions.

The results obtained from the MS identification of the differentially expressed protein profiles in the control and experimental samples are presented below.  Figure 3 shows a Venn diagram that illustrates the overlaps and differences in the protein profile of the investigated strains, treated and untreated with 0.20% CHX.

A total of 169 different proteins were identified, the listing of all the different proteins expressed by both the *P. gingivalis* cells that were not exposed to CHX (control) and the bacterial cells that were exposed to 0.20% CHX (experimental) can be found in the File S1. Forty-three proteins were identified in the control group, of which seven proteins were unique in that group, while in the experimental group (bacterial cells treated with 0.20% CHX), 126 proteins were identified, of which 90 different proteins were unique to that group. Finally, 36 proteins were identified in both the control and experimental groups.  Table 1 lists the 20 more expressed proteins by both the control group and the experimental group. The table lists the 10 proteins with the highest expression in the control and experimental groups. For a complete list of the 169 different proteins expressed by both the cells that were not exposed to CHX (control) and the bacterial cells that were exposed to 0.20% CHX (experimental).

From the protein extracts obtained from bacterial cells exposed to 0.20% CHX the proteins that had the greatest expression were, first, Lys gingipain, followed by outer membrane protein 41, peptidoglycan domain protein, and outer membrane protein 40, all followed in degree of expression by co chaperonin GroES.

On the other hand, organized also based on their expression, from higher to lower, we can see that within the proteins that showed the highest expression by bacterial cells that were not exposed to CHX (control) are in the first place Lys gingipain, followed by Arg gingipain, then, superoxide dismutase (SOD) protein, followed by DNA binding protein and 50S ribosomal protein L31 type B.

An important finding from the results of protein identification by MS is that within the group of proteins that were expressed in both the control and experimental groups, changes in the expression of those proteins were observed, both increase and decrease in their expression depending on whether the bacterial cells were exposed to 0.20% CHX or not.  Table 2 lists the 36 different proteins that were expressed by both the control group and the experimental group.

From the above table, we can observe that the proteins that had the greatest increases in their expression by cause of the exposure of *P. gingivalis* to 0.20% CHX were outer membrane protein 41, 30S ribosomal protein S6, 50S ribosomal protein L3, and TPR domain protein, all of them had an increase in their expression by more than six times. It is worth noting that protein 41 of the outer membrane had a ninefold increase in its expression, since it is a protein that has functions of noncovalent interactions with the bacterial peptidoglycan, this increase confirms that the main mechanism of action of 0.20% CHX is through direct damage to the bacterial wall.

## 4. Discussion

*Porphyromonas gingivalis* plays a key role in the pathogenesis and severity of periodontitis by disrupting the balance between symbionts and pathobionts of the oral microbiota that results in biofilm dysbiosis [30, 31]. Further, it modulates the host immune response to give itself and other biofilm constituents an advantage to thrive and make the host more vulnerable to infection [32]; therefore, chemical compounds such as 0.20% CHX have historically been used to reduce their periodontal load [23]. 0.20% CHX use is advised, not only for the control of dental biofilm but also for dental implantology as it significantly reduces the host's inflammatory response around implants by significantly reducing *P. gingivalis* load [33]. Due to the important role that CHX plays in reducing the oral bacterial load; in the present work, we sought to determine how exposure to 0.20% CHX affects the growth and the protein expression of *P. gingivalis*, thus, corroborating a previously reported mechanism of action on bacterial cell wall damage, which results in the leakage of cytoplasmic constituents and subsequent coagulation of the cytoplasm, as observed through transmission microscopy images [19].

From the present study, several interesting observations were made. First, 0.20% CHX exerted approximately 93% inhibitory effect on *P. gingivalis* after 1 min of exposure, this result is in line with a previous report that showed that under in vivo conditions, the use of a 0.20% CHX as a mouthwash for 30 s can induce bacterial inhibition of the salivary microbiota by approximately 90% [34]. In addition, another study reported that one mouth rinse with 0.20% CHX per day for 1 min resulted in a significant reduction of the total oral planktonic microbiota [35].

**Figure 4:**
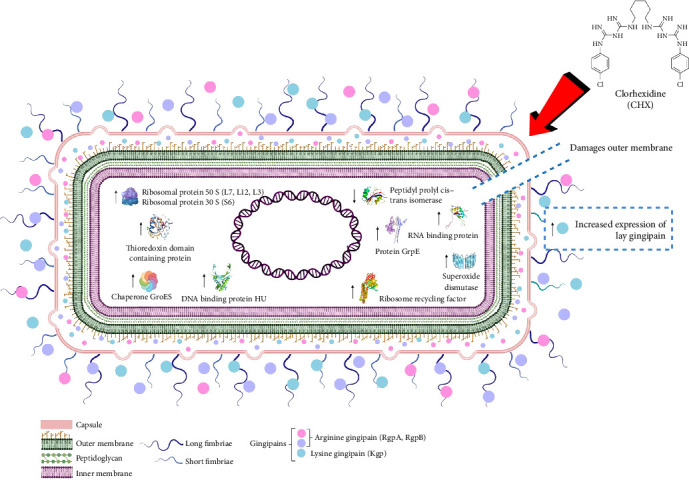
Illustration of the main changes in the proteome of *Porphyromonas gingivalis* derived from their exposure during 1 min to 0.20% CHX. Mainly, increased expression of ribosomal protein 30S and 50S, thioredoxin domain contain protein, chaperone GroEs, RNA binding protein, and protein GrpE. Whereas decreased expression of peptidyl prolyl cis–trans isomerase.

Another important finding was the identification of specific changes in the proteome of *P. gingivalis* derived from their brief exposure to 0.20% CHX (Figure 4). It was found that the protein with the higher expression both by bacterial cells exposed to 0.20% CHX and by those *P. gingivalis* cells not treated (control) was lysine gingipain, this indicated that *P. gingivalis* consistently expresses this important factor of virulence regardless of whether it is exposed to a harmful stimulus, such as CHX.

Gingipains constitute a group of cysteine endopeptidases that are responsible for at least 100% of the so-called “trypsin-like activity” produced by *P. gingivalis* [36, 37]. Lysine gingipain is an extracellular protein encoded by the *kgp* gene that can bind to red blood cells and heme-containing proteins, including hemoglobin, these proteases act as a hemolytic enzyme to sequester and store iron, which is an essential factor in growth, survival, and function of *P. gingivalis* [38]. Therefore, the increase in the expression of lysine gingipain found in the present work could be expected since bacteria use such enzyme to protect themselves against severe redox stress and restricted availability of iron in the human body or in the culture medium, according to the experimental conditions under which the bacteria was grown in this research. *Porphyromonas gingivalis* overexpressing Lys gingipain after a brief exposure to 0.20% CHX should, however, be further investigated, given that the expression of this important virulence factor is related to immunosuppressive effects, in addition to being involved in coaggregation processes with other pathogenic periodontal bacteria such as *Treponema denticola* [10, 39].

In addition to the predominance in gingipain expression, in the control group (bacterial cells not exposed to 0.20% CHX) the expression of the protease PrtH was identified, this protease is normally found in membrane vesicles produced by *P. gingivalis* and can bind to complement protein C3 under defined conditions [40]. Besides, SOD, an enzyme that was expressed by both the control and experimental groups, catalyzes the dismutation of superoxide and is considered important for the protection of cells against toxic superoxide anion, this finding is meaningful given that it has been identified that the expression of this enzyme increases up to three times when *P. gingivalis* is exposed to stimuli such as temperature increase (from 37 to 39°C) [37], in the present research an increase of more than twofold was observed in the bacterial cells that were exposed to CHX, experimental group, comparing to the control group.

It has been previously reported that the heat shock response is highly conserved in both eukaryotic and prokaryotic cells and is clustered in two major molecular chaperone protein families, DnaK (HSP70) and GroEL (HSP60), which are involved in protein folding, oligomerization, translocation, and degradation [37, 41]. In the present research work, the expression of GroES, GroEL, and DnaK chaperones was found in the experimental group, given the protective function of these proteins, their identification in the group of bacteria that were exposed to 0.20% CHX was a coherent finding. Importantly, a 4.8-fold increase in the expression of the chaperone GroES in the experimental group compared to the control was observed, this would indicate that the exposure of *P. gingivalis* to 0.20% CHX for a brief period (1 min) can induce the denaturation of bacterial cellular proteins and the increase in the expression of this chaperone would reflect an attempt to counteract such denaturation by refolding of the affected proteins [37, 42].

At the molecular level, the mechanism by which CHX acts on oral biofilm should be elucidated in further studies, as antimicrobials act differently depending on whether the bacteria are in a planktonic state or whether they are in a biofilm [43]. Biofilm is the preferred mode of bacterial growth in nature. It confers many benefits, such as increased ability to withstand the immune response and increased resistance to antibiotics and antiseptics [44].

It has been reported that oral bacteria, such as *Streptococcus sanguinis* and *P. gingivalis*, are more susceptible to CHX in the planktonic state than in the biofilm state [45–47], because antimicrobials agents such as CHX are inactivated by extracellular polysaccharides (EPSs) produced by bacteria within the biofilm [48]. Cationic compounds such as CHX undergo electrostatic interactions and sorption with the negatively charged EPS structure, which limits their penetration into the biofilm structure [49]. On this matter, a previous laser scanning optical confocal microscopy study showed that treatment with 0.2% CHX for 1 min only affected the outer layers of biofilms formed in situ for 48 h [50]. It is estimated that antiseptics concentrations required to kill sessile bacterial cells in biofilms are approximately 100–1000 times higher than those needed to kill planktonic bacterial cells [51].

In conclusion, the results obtained in the present work confirmed the in vitro inhibitory effect of 0.20% CHX on *P. gingivalis* in a planktonic state. In addition, it was possible to identify that the changes in the proteome of *P. gingivalis* after exposure to 0.20% CHX resulted in an approximately 2.9-fold increase in protein expression.

The results derived from the protein identification indicate that the response of *P. gingivalis* to 0.20% CHX exposure is characterized by an increase of approximately 2.6 times in the expression of the lysine gingipain protease, which indicates the extracellular proteolytic activity of *P. gingivalis* executed for its survival against a harmful stimulus; in addition, it could indicate mechanisms of bacterial resistance, the latter should be explored further in future research. Importantly, the approximately 4.8-fold increase in GroES chaperone expression would indicate that one of the major mechanisms of action of CHX on *P. gingivalis* is through the denaturation of bacterial cell proteins.

## Figures and Tables

**Table 1:** The table lists the 10 proteins with the highest expression in the control and experimental groups.

Experimental				
#	Accession	Description	mW (kDa)	Function
1	KGP_PORG3	Lys gingipain	187.1	Cysteine-type endopeptidase activity
2	B2RMB8_PORG3	Uncharacterized protein	10.3	Unknown
3	B2RIQ3_PORG3	Outer membrane protein 41	43.3	Non-covalent interactions with peptidoglycan
4	A0A2D2P334_PORGN	Peptidoglycan domain protein	21.9	Peptidoglycan binding
5	B2RIQ2_PORG3	Outer membrane protein 40	42.4	Non-covalent interactions with peptidoglycan
6	A0A212G636_PORGN	Co chaperonin GroES	14.1	ATP-dependent protein folding chaperone
7	B2RHA9_PORG3	DNA binding protein HU	9.4	Structural constituent of chromatin
8	B2RI88_PORG3	Superoxide dismutase	21.4	Superoxide dismutase activity
9	B2RH30_PORG3	OMP b brl 2 domain containing protein	24.3	Transport of metabolites and toxins
10	B2RJ50_PORG3	TPR domain protein	45.7	Mediate protein–protein interactions and the assembly of multiprotein complexes

**Control**				
**#**	**Accession**	**Description**	**mW (kDa)**	**Function**

1	KGP_PORG3	Lys gingipain	187.1	Cysteine-type endopeptidase activity
2	Q51816_PORGN	Arg gingipain 1 proteinase	185.3	Cysteine-type endopeptidase activity
3	A0A0K2J6P1_PORGN	Uncharacterized protein	184.7	Unknown
4	B2RI88_PORG3	Superoxide dismutase	21.4	Superoxide dismutase activity
5	B2RHA9_PORG3	DNA binding protein HU	9.4	Structural constituent of chromatin
6	A0A212G9N0_PORGN	Uncharacterized protein	9.4	Unknown
7	A0A212G7L4_PORGN	50S ribosomal protein L31 type B	9.7	Protein synthesis
8	B2RL53_PORG3	Putative site-specific recombinase	46.3	DNA binding
9	PRTH_PORGI	Protease PrtH	110.1	Cysteine-type peptidase activity
10	B2RIQ2_PORG3	Outer membrane protein 40	42.4	Noncovalent interactions with peptidoglycan

*Note*: For a complete list of the 169 different proteins expressed by both the cells that were not exposed to CHX (control) and the bacterial cells that were exposed to 0.20% CHX (experimental) please refer to the File S1.

Abbreviation: CHX, chlorhexidine.

**Table 2:** Lists 36 different proteins that were expressed both by bacterial cells that were exposed to 0.20% CHX (experimental) and by *Porphyromonas gingivalis* cells that were not treated with CHX for 1 min.

#	Accession	Description	mW (kDa)	Function	Changes in their expression
1	B2RIQ3_PORG3	Outer membrane protein 41	43.3	Noncovalent interactions with peptidoglycan	9 **↑**
2	A0A134DPK6_PORGN	30S ribosomal protein S6	13.5	Protein synthesis	7 **↑**
3	A0A212G9N0_PORGN	Uncharacterized protein	9.4	Unknown	7 **↑**
4	A0A212GB86_PORGN	50S ribosomal protein L3	20.8	Protein synthesis	6.5 **↑**
5	B2RJ50_PORG3	TPR domain protein	45.7	Mediate protein–protein interactions and the assembly of multiprotein complexes	6.25 **↑**
6	B2RHR2_PORG3	Thiol peroxidase	19	Thioredoxin peroxidase activity	6 ↑
7	A0A212G879_PORGN	2-oxoglutarate ferredoxin oxidoreductase subunit gamma	20.1	Oxidoreductase activity	6 **↑**
8	A0A134DNY8_PORGN	DUF3467 domain containing protein	11.8	Unknown	5 ↑
9	A0A212G7Z9_PORGN	Co chaperonin GroES	9.6	ATP-dependent protein folding chaperone	4.75 ↑
10	A0A212G983_PORGN	Cell division protein	18.6	Peptidoglycan binding	4.6 ↑
11	B2RHG7_PORG3	Receptor antigen A	114.4	Receptor	4 **↑**
12	B2RHA9_PORG3	DNA binding protein HU	9.4	Structural constituent of chromatin	4 **↑**
13	B2RHM3_PORG3	Upregulated in stationary phase protein A	9	Protein growth ceases but cells remain metabolically active	3.8 **↑**
14	A0A212G9K0_PORGN	Electron transfer flavoprotein subunit beta	28.5	Electron transfer activity	3.75 **↑**
15	B2RH12_PORG3	Uncharacterized protein	15.7	Unknown	3.5 **↑**
16	A0A212G8M5_PORGN	Protein GrpE	21.7	Protein-folding chaperone binding	3.3 **↑**
17	A0A212G987_PORGN	Uncharacterized protein	8.7	Unknown	3.25 **↑**
18	Q7MWI3_PORGI	RNA binding protein	11.4	RNA binding	2.6 **↑**
19	B2RIQ2_PORG3	Outer membrane protein 40	42.4	Noncovalent interactions with peptidoglycan	2.6 **↑**
20	KGP_PORG3	Lys gingipain	187.1	Cysteine-type endopeptidase activity	2.6 **↑**
21	B2RI27_PORG3	Putative biotin carboxyl carrier protein	15.2	Acetyl-CoA carboxylase activity	2.5 **↑**
22	A0A212G7L4_PORGN	50S ribosomal protein L31 type B	9.7	Protein synthesis	2.5 **↑**
23	A0A134DQU1_PORGN	Membrane protein	19.6	Unfolded protein binding	2.4 **↑**
24	A0A212G9M3_PORGN	VOC domain containing protein	14.7	Metalloenzyme activity	2.25 **↑**
25	A0A212GCC1_PORGN	50S ribosomal protein L7 L12	12.6	Protein synthesis	2.2 **↑**
26	B2RI88_PORG3	Superoxide dismutase (SOD)	21.4	SOD activity	2.1 **↑**
27	A0A1R4DT75_PORGN	Protein TonB	49	Energy transducer activity	2 **↑**
28	B2RHG8_PORG3	Receptor antigen B	56.8	Receptor	2 **↑**
29	B2RGZ4_PORG3	Thioredoxin domain containing protein	17.7	Oxidoreductase activity	2 **↑**
30	A0A134DMK3_PORGN	Acyl carrier protein	8.7	Cofactor of both fatty acid and polyketide biosynthesis machinery	2 **↑**
31	A0A212G0V8_PORGN	Ribosome recycling factor	20.7	Release of ribosomes from messenger RNA at the termination of protein biosynthesis	2 **↑**
32	B2RMB8_PORG3	Uncharacterized protein	10.3	Unknown	1.8 **↑**
33	B2RL53_PORG3	Putative site specific recombinase	46.3	DNA binding	1.6 **↑**
34	B2RKW2_PORG3	DUF5606 domain containing protein	16.2	Unknown	1.5 **↑**
35	B2RIR7_PORG3	Peptidyl prolyl cis trans isomerase	28.4	Peptidyl-prolyl cis-trans isomerase activity	1.25 **↓**
36	B2RGQ7_PORG3	Thioredoxin	11.4	Protein-disulfide reductase activity	** = **

*Note:* Based on *P. gingivalis* exposure to 0.20% CHX, the right column indicates how often a protein changed its overexpression (**↑** up arrows) or downexpression (**↓** down arrow).

## Data Availability

All data on the results of this study are included in this document and its supporting information files. The corresponding author is the owner of this document and of the data presented in it.

## Supporting Information

Supporting Information File S1: table listing the total of 169 different proteins expressed by both the *Porphyromonas gingivalis* cells that were not exposed to chlorhexidine (control) and the bacterial cells that were exposed to 0.20% CHX (experimental). The proteins listed in the table were organized according to their expression, from highest to lowest.
