# Supplementary material for: Insights Into the Mechanism of Action of Chlorhexidine on Porphyromonas gingivalis
Source: Int J Dent. 2025 Feb 27;2025:1492069. doi: 10.1155/ijod/1492069 (PMC11986949; doi:10.1155/ijod/1492069)
Supplement: Supporting Information — File S1: table listing the total of 169 different proteins expressed by both the Porphyromonas gingivalis cells that were not exposed to chlorhexidine (control) and the bacterial cells that were exposed to 0.20% CHX (experimental). The proteins listed in the table were organized according to their expression, from highest to lowest. [file 1492069.f1.docx]

***Supplementary file.*** Table listing the total of 169 different proteins expressed by both the *P. gingivalis* cells that were not exposed to chlorhexidine (**control**) and the bacterial cells that were exposed to 0.20% CHX (**experimental**). The proteins listed in the table were organized according to their expression, from highest to lowest.

| **CONTROL** | | | | |
| --- | --- | --- | --- | --- |
| **#** | **Accession** | **Description** | **mW (kDa)** | **Function** |
| 1 | KGP_PORG3 | Lys gingipain | 187,1 | Cysteine-type endopeptidase activity |
| 2 | Q51816_PORGN | Arg gingipain 1 proteinase | 185,3 | Cysteine-type endopeptidase activity |
| 3 | A0A0K2J6P1_PORGN | Uncharacterized protein | 184,7 | Unknown |
| 4 | B2RI88_PORG3 | Superoxide dismutase | 21,4 | Superoxide dismutase activity |
| 5 | B2RHA9_PORG3 | DNA binding protein HU | 9,4 | Structural constituent of chromatin |
| 6 | A0A212G9N0_PORGN | Uncharacterized protein | 9,4 | Unknown |
| 7 | A0A212G7L4_PORGN | 50S ribosomal protein L31 type B | 9,7 | Protein synthesis |
| 8 | B2RL53_PORG3 | Putative site specific recombinase | 46,3 | DNA binding |
| 9 | PRTH_PORGI | Protease PrtH | 110,1 | Cysteine-type peptidase activity |
| 10 | B2RIQ2_PORG3 | Outer membrane protein 40 | 42,4 | Non-covalent interactions with peptidoglycan |
| 11 | B2RMB8_PORG3 | Uncharacterized protein | 10,3 | Unknown |
| 12 | A0A212GCC1_PORGN | 50S ribosomal protein L7 L12 | 12,6 | Protein synthesis |
| 13 | B2RGZ4_PORG3 | Thioredoxin domain containing protein | 17,7 | Oxidoreductase activity |
| 14 | Q9RHI2_PORGN | Elongation factor Tu | 43,6 | Translation elongation factor activity |
| 15 | B2RGQ7_PORG3 | Thioredoxin | 11,4 | Protein-disulfide reductase activity |
| 16 | A0A212GAZ4_PORGN | Uncharacterized protein | 5,5 | Unknown |
| 17 | A0A212G0V8_PORGN | Ribosome recycling factor | 20,7 | Release of ribosomes from messenger RNA at the termination of protein biosynthesis. |
| 18 | B2RHG7_PORG3 | Receptor antigen A | 114,4 | Receptor |
| 19 | Q7MWI3_PORGI | RNA binding protein | 11,4 | RNA binding |
| 20 | B2RKW2_PORG3 | DUF5606 domain containing protein | 16,2 | Unknown |
| 21 | T2NDG7_PORGN | Cleaved adhesin domain protein Fragment | 37 | Cysteine-type peptidase activity |
| 22 | A0A212G987_PORGN | Uncharacterized protein | 8,7 | Unknown |
| 23 | A0A134DMK3_PORGN | Acyl carrier protein | 8,7 | Cofactor of both fatty acid and polyketide biosynthesis machinery. |
| 24 | B2RHG8_PORG3 | Receptor antigen B | 56,8 | Receptor |
| 25 | B2RHM3_PORG3 | Upregulated in stationary phase protein A | 9 | Protein growth ceases but cells remain metabolically active |
| 26 | A0A212G7Z9_PORGN | Co chaperonin GroES | 9,6 | ATP-dependent protein folding chaperone |
| 27 | B2RI27_PORG3 | Putative biotin carboxyl carrier protein | 15,2 | Acetyl-CoA carboxylase activity |
| 28 | A0A134DQU1_PORGN | Membrane protein | 19,6 | Unfolded protein binding |
| 29 | B2RIR7_PORG3 | Peptidyl prolyl cis trans isomerase | 28,4 | Peptidyl-prolyl cis-trans isomerase activity |
| 30 | A0A134DNY8_PORGN | DUF3467 domain containing protein | 11,8 | Unknown |
| 31 | A0A212G9M3_PORGN | VOC domain containing protein | 14,7 | Metalloenzyme activity |
| 32 | A0A212GB86_PORGN | 50S ribosomal protein L3 | 20,8 | Protein synthesis |
| 33 | B2RIQ3_PORG3 | Outer membrane protein 41 | 43,3 | Non-covalent interactions with peptidoglycan |
| 34 | A0A212G983_PORGN | Cell division protein | 18,6 | Peptidoglycan binding |
| 35 | A0A1R4DT75_PORGN | Protein TonB | 49 | Energy transducer activity |
| 36 | A0A212G8M5_PORGN | Protein GrpE | 21,7 | Protein-folding chaperone binding |
| 37 | B2RHR2_PORG3 | Thiol peroxidase | 19 | Thioredoxin peroxidase activity |
| 38 | A0A134DPK6_PORGN | 30S ribosomal protein S6 | 13,5 | Protein synthesis |
| 39 | A0A212G879_PORGN | 2 oxoglutarate ferredoxin oxidoreductase subunit gamma | 20,1 | Oxidoreductase activity |
| 40 | B2RH12_PORG3 | Uncharacterized protein | 15,7 | Unknown |
| 41 | B2RJ50_PORG3 | TPR domain protein | 45,7 | Mediate protein-protein interactions and the assembly of multiprotein complexes |
| 42 | A0A212G9K0_PORGN | Electron transfer flavoprotein subunit beta | 28,5 | Electron transfer activity |
| 43 | A0A1D3UQ75_TANFO | Bacterial Ig like domain Group 2 | 58,4 | Mediates the bacterial host-cell interaction |
| **EXPERIMENTAL** | | | | |
| 1 | KGP_PORG3 | Lys gingipain | 187,1 | Cysteine-type endopeptidase activity |
| 2 | B2RMB8_PORG3 | Uncharacterized protein | 10,3 | Unknown |
| 3 | B2RIQ3_PORG3 | Outer membrane protein 41 | 43,3 | Non-covalent interactions with peptidoglycan |
| 4 | A0A2D2P334_PORGN | Peptidoglycan domain protein | 21,9 | Peptidoglycan binding |
| 5 | B2RIQ2_PORG3 | Outer membrane protein 40 | 42,4 | Non-covalent interactions with peptidoglycan |
| 6 | A0A212G636_PORGN | Co chaperonin GroES | 14,1 | ATP-dependent protein folding chaperone |
| 7 | B2RHA9_PORG3 | DNA binding protein HU | 9,4 | Structural constituent of chromatin |
| 8 | B2RI88_PORG3 | Superoxide dismutase | 21,4 | Superoxide dismutase activity |
| 9 | B2RH30_PORG3 | OMP b brl 2 domain containing protein | 24,3 | Transport of metabolites and toxins |
| 10 | B2RJ50_PORG3 | TPR domain protein | 45,7 | Mediate protein-protein interactions and the assembly of multiprotein complexes |
| 11 | B2RL53_PORG3 | Putative site specific recombinase | 46,3 | DNA binding |
| 12 | A0A134DNY8_PORGN | DUF3467 domain containing protein | 11,8 | Unknown |
| 13 | A0A212GCC1_PORGN | 50S ribosomal protein L7 L12 | 12,6 | Protein synthesis |
| 14 | A0A134DQU1_PORGN | Membrane protein | 19,6 | Unfolded protein binding |
| 15 | A0A212G9N0_PORGN | Uncharacterized protein | 9,4 | Unknown |
| 16 | A0A212G0V8_PORGN | Ribosome recycling factor | 20,7 | Release of ribosomes from messenger RNA at the termination of protein biosynthesis. |
| 17 | A0A212G987_PORGN | Uncharacterized protein | 8,7 | Unknown |
| 18 | A0A212G7L4_PORGN | 50S ribosomal protein L31 type B | 9,7 | Protein synthesis |
| 19 | A0A212GB59_PORGN | 50S ribosomal protein L24 | 11,5 | Protein synthesis |
| 20 | A0A212G879_PORGN | 2 oxoglutarate ferredoxin oxidoreductase subunit gamma | 20,1 | Oxidoreductase activity |
| 21 | B2RHM3_PORG3 | Upregulated in stationary phase protein A | 9 | Protein growth ceases but cells remain metabolically active |
| 22 | B2RI27_PORG3 | Putative biotin carboxyl carrier protein | 15,2 | Acetyl-CoA carboxylase activity |
| 23 | B2RHG7_PORG3 | Receptor antigen A | 114,4 | Receptor |
| 24 | A0A0E2LNN4_PORGN | Putative DNA binding protein | 18,2 | DNA binding |
| 25 | A0A212G715_PORGN | DNA binding protein | 10,2 | Structural constituent of chromatin |
| 26 | B2RK03_PORG3 | Phosphate acetyltransferase | 35,8 | Acetyltransferase activity |
| 27 | A0A212G983_PORGN | Cell division protein | 18,6 | Peptidoglycan binding |
| 28 | A0A212G8M5_PORGN | Protein GrpE | 21,7 | Protein-folding chaperone binding |
| 29 | A0A212G9K0_PORGN | Electron transfer flavoprotein subunit beta | 28,5 | Electron transfer activity |
| 30 | A0A212G8A3_PORGN | Ferredoxin | 8,1 | Iron-sulfur cluster binding |
| 31 | A0A134DMK3_PORGN | Acyl carrier protein | 8,7 | Cofactor of both fatty acid and polyketide biosynthesis machinery. |
| 32 | A0A212G7Z9_PORGN | Co chaperonin GroES | 9,6 | ATP-dependent protein folding chaperone |
| 33 | B2RHY8_PORG3 | Uncharacterized protein | 112,3 | Unknown |
| 34 | A0A1R4DUR8_PORGN | Malate dehydrogenase | 47,2 | Malate dehydrogenase activity |
| 35 | B2RGQ7_PORG3 | Thioredoxin | 11,4 | Protein-disulfide reductase activity |
| 36 | Q9RHI0_PORGN | Elongation factor Tu | 43,6 | Translation elongation factor activity |
| 37 | B2RHR2_PORG3 | Thiol peroxidase | 19 | Thioredoxin peroxidase activity |
| 38 | A0A212G8U9_PORGN | Uncharacterized protein | 52 | Unknown |
| 39 | A0A134DPK6_PORGN | 30S ribosomal protein S6 | 13,5 | Protein synthesis |
| 40 | B2RKW2_PORG3 | DUF5606 domain containing protein | 16,2 | Unknown |
| 41 | B2RMC1_PORG3 | Immunoreactive 23 kDa antigen | 22,5 | Reacts to a particular antigen |
| 42 | A0A212G4Z6_PORGN | Multifunctional fusion protein | 107,1 | Protein-transporting ATPase activity |
| 43 | A0A212G9U6_PORGN | 2 3 bisphosphoglycerate dependent phosphoglycerate mutase | 28,7 | 2,3-bisphosphoglycerate-dependent phosphoglycerate mutase activity |
| 44 | A0A212G713_PORGN | Serine hydroxymethyltransferase | 46,6 | Methyltransferase activity |
| 45 | B2RGZ4_PORG3 | Thioredoxin domain containing protein | 17,7 | Oxidoreductase activity |
| 46 | B2RK30_PORG3 | Bifunctional protein FolD | 31,9 | Methenyltetrahydrofolate cyclohydrolase activity |
| 47 | B2RIG1_PORG3 | Putative heat shock related protease htrA protein | 52,8 | Metalloendopeptidase activity |
| 48 | A0A212G1A0_PORGN | Phosphate acetyltransferase | 43,2 | Acetyltransferase activity |
| 49 | A0A212GC03_PORGN | Uncharacterized protein | 62,4 | Unknown |
| 50 | A0A212G7N1_PORGN | Indolepyruvate oxidoreductase | 21 | Oxidoreductase activity |
| 51 | A0A212G9M3_PORGN | VOC domain containing protein | 14,7 | Metalloenzyme activity |
| 52 | A0A212GB86_PORGN | 50S ribosomal protein L3 | 20,8 | Protein synthesis |
| 53 | DNAK_PORG3 | Chaperone protein DnaK | 69 | ATP-dependent protein folding chaperone |
| 54 | A0A829KLT7_PORGN | Tetratricopeptide repeat protein | 112,2 | Protein import |
| 55 | B2RLH8_PORG3 | 3 oxoacyl acyl carrier protein synthase 2 | 44,4 | 3-oxoacyl-[acyl-carrier-protein] synthase activity |
| 56 | A0A212GC43_PORGN | 50S ribosomal protein L11 | 15,5 | Protein synthesis |
| 57 | A0A212GA93_PORGN | zf RING 7 domain containing protein | 28,7 | Protein ligase |
| 58 | B2RGV7_PORG3 | Uncharacterized protein | 52,7 | Unknown |
| 59 | PCKA_PORG3 | Phosphoenolpyruvate carboxykinase ATP | 59,3 | Phosphoenolpyruvate carboxykinase (ATP) activity |
| 60 | Q7MWI3_PORGI | RNA binding protein | 11,4 | RNA binding |
| 61 | B2RIW2_PORG3 | Peptidyl dipeptidase | 79,4 | Metalloendopeptidase activity |
| 62 | B2RM28_PORG3 | Hemagglutinin protein HagB | 39,3 | Adhesin |
| 63 | B2RI40_PORG3 | Putative DnaK suppressor protein | 14,3 | Zinc ion binding |
| 64 | B2RHC5_PORG3 | Malonyl CoA acyl carrier protein transacylase | 31,4 | [acyl-carrier-protein] S-malonyltransferase activity |
| 65 | A0A134DNY2_PORGN | RNA polymerase Rpb6 | 12,9 | DNA binding |
| 66 | A0A212GB83_PORGN | 50S ribosomal protein L29 | 7,4 | Protein synthesis |
| 67 | B2RII4_PORG3 | Alkyl hydroperoxide reductase C | 21 | Plays a role in cell protection against oxidative stress by detoxifying peroxides. |
| 68 | A0A1D3UME7_TANFO | 50S ribosomal protein L3 | 21,9 | Protein synthesis |
| 69 | B2RKY4_PORG3 | Putative peptidyl prolyl cis trans isomerase | 79,4 | Peptidyl-prolyl cis-trans isomerase activity |
| 70 | B2RIX5_PORG3 | Probable electron transfer flavoprotein beta subunit | 30,9 | Electron transfer activity |
| 71 | B2RLR5_PORG3 | Flavodoxin | 17,1 | Electron transfer activity |
| 72 | B2RHH5_PORG3 | Uncharacterized protein | 18,7 | Unknown |
| 73 | A0A212G7Q7_PORGN | NADP oxidoreductase | 41,2 | Oxidoreductase activity |
| 74 | B2RHG8_PORG3 | Receptor antigen B | 56,8 | Receptor |
| 75 | A0A134DRV6_PORGN | 30S ribosomal protein S2 | 31,3 | Protein synthesis |
| 76 | B2RJR5_PORG3 | Uncharacterized protein | 23,2 | Unknown |
| 77 | A0A212G7R1_PORGN | 50S ribosomal protein L9 | 19,4 | Protein synthesis |
| 78 | B2RH02_PORG3 | Immunoreactive 53 kDa antigen | 53,5 | Reacts to a particular antigen |
| 79 | A0A2A6EIW8_PREIN | Uncharacterized protein | 52,5 | Unknown |
| 80 | A0A212GAQ3_PORGN | 30S ribosomal protein S16 | 20,6 | Protein synthesis |
| 81 | B2RLM9_PORG3 | Putative 2 oxoglutarate oxidoreductase beta subunit | 25,3 | Thiamine pyrophosphate binding |
| 82 | A0A212G644_PORGN | Uncharacterized protein | 23,2 | Unknown |
| 83 | A0A212GAW2_PORGN | Aminopeptidase | 53 | Unknown |
| 84 | A0A134DPL6_PORGN | Aspartyl tRNA amidotransferase subunit B | 16,3 | Transferase activity |
| 85 | A0A212FQU6_PORGN | Adenylosuccinate synthetase | 46,5 | Catalyzes the first committed step in the biosynthesis of AMP from IMP. |
| 86 | B2RHL9_PORG3 | Uncharacterized protein | 32 | Unknown |
| 87 | B2RH12_PORG3 | Uncharacterized protein | 15,7 | Unknown |
| 88 | B2RJL1_PORG3 | Uncharacterized protein | 15,5 | Unknown |
| 89 | A0A134DR96_PORGN | 50S ribosomal protein L18 | 12,5 | Protein synthesis |
| 90 | A0A212GB65_PORGN | 30S ribosomal protein S12 | 14,7 | Protein synthesis |
| 91 | B2RIN4_PORG3 | Indolepyruvate oxidoreductase subunit IorA | 58,3 | Indolepyruvate ferredoxin oxidoreductase activity |
| 92 | A0A1D3UKP3_TANFO | Ribosome recycling factor | 20,8 | Release of ribosomes from messenger RNA at the termination of protein biosynthesis. |
| 93 | A0A2A6E8G4_TANFO | Serine hydroxymethyltransferase | 46,5 | Methyltransferase activity |
| 94 | A0A134DPN1_PORGN | 30S ribosomal protein S18 | 10,6 | Protein synthesis |
| 95 | A0A0K2J4B1_PORGN | Glutamate dehydrogenase | 48,4 | Glutamate dehydrogenase [NAD(P)+] activity |
| 96 | B2RJC6_PORG3 | Carboxyl terminal processing protease | 63,4 | Metalloendopeptidase activity |
| 97 | A0A212G3Y8_PORGN | 50S ribosomal protein L30 | 6,8 | Protein synthesis |
| 98 | A0A212GC72_PORGN | Elongation factor Ts | 30,1 | Translation elongation factor activity |
| 99 | B2RHU8_PORG3 | Putative HIT family protein | 14,3 | Catalytic activity |
| 100 | B2RIY3_PORG3 | Putative TonB protein | 25,7 | Receptor |
| 101 | A0A2D2N416_PORGN | OmpA like domain containing protein | 53,5 | Non-covalent interactions with peptidoglycan |
| 102 | A0A1D3ULR7_TANFO | Chaperone protein Skp | 19,7 | Unfolded protein binding |
| 103 | B2RIR7_PORG3 | Peptidyl prolyl cis trans isomerase | 28,4 | Peptidyl-prolyl cis-trans isomerase activity |
| 104 | B2RLA4_PORG3 | Transcription termination factor Rho | 72 | ATP-dependent activity, acting on RNA |
| 105 | A0A212G7Z3_PORGN | Chaperonin GroEL | 58,1 | ATP-dependent protein folding chaperone |
| 106 | A0A134DRQ5_PORGN | 50S ribosomal protein L21 | 11,8 | Protein synthesis |
| 107 | A0A212G8S7_PORGN | Uncharacterized protein | 20,6 | Unknown |
| 108 | A0A212GA95_PORGN | 4 hydroxybutyryl CoA dehydratase | 53,9 | Oxidoreductase activity |
| 109 | B2RJW7_PORG3 | Uncharacterized protein | 16,3 | Unknown |
| 110 | B2RGP7_PORG3 | Por secretion system protein porV Pg27 lptO | 43,2 | Secretion of major extracellular proteinases |
| 111 | A0A212G971_PORGN | Polyketide cyclase | 15,3 | Polyketide synthesis |
| 112 | A0A134DQ06_PORGN | Glycine cleavage system H protein | 13,8 | Catalyzes the degradation of glycine |
| 113 | A0A212GBE8_PORGN | 50S ribosomal protein L15 | 15,5 | Protein synthesis |
| 114 | B2RKE5_PORG3 | Uncharacterized protein | 50 | Unknown |
| 115 | B2RJY8_PORG3 | Uncharacterized protein | 29,7 | Unknown |
| 116 | A0A134DQE1_PORGN | Aspartate 1 decarboxylase | 12,8 | Catalyzes the pyruvoyl-dependent decarboxylation of aspartate to produce beta-alanine. |
| 117 | B2RK80_PORG3 | S4 domain containing protein | 17,5 | RNA binding |
| 118 | Q7MVY9_PORGI | Bacterioferritin comigratory protein | 19 | Peroxidase activity |
| 119 | A0A212G330_PORGN | Elongation factor P | 21,1 | Translation elongation factor activity |
| 120 | A0A212G6L3_PORGN | Histidinol phosphate aminotransferase | 17 | Transaminase activity |
| 121 | A0A212GAB7_PORGN | Peptidyl prolyl cis trans isomerase | 29 | Peptidyl-prolyl cis-trans isomerase activity |
| 122 | T2NB07_PORGN | Tetratricopeptide repeat protein | 132,2 | Protein import |
| 123 | A0A212G799_PORGN | Uncharacterized protein | 65,3 | Unknown |
| 124 | B2RLA7_PORG3 | Glutamate formimidoyltransferase | 33,1 | Glutamate formimidoyltransferase activity |
| 125 | A0A1R4DT75_PORGN | Protein TonB | 49 | Energy transducer activity |
| 126 | A0A2D2N4K1_PORGN | Peptidase M13 | 78,8 | Metalloendopeptidase activity |
